# Traditional Chinese Medicine and Gut Microbiome: Their Respective and Concert Effects on Healthcare

**DOI:** 10.3389/fphar.2020.00538

**Published:** 2020-04-22

**Authors:** Runzhi Zhang, Xi Gao, Hong Bai, Kang Ning

**Affiliations:** School of Life Science and Technology, Huazhong University of Science and Technology, Wuhan, China

**Keywords:** traditional Chinese medicine, gut microbiome, healthcare, concert effects, tools and databases

## Abstract

Advances in systems biology, particularly based on the omics approaches, have resulted in a paradigm shift in both traditional Chinese medicine (TCM) and the gut microbiome research. In line with this paradigm shift, the importance of TCM and gut microbiome in healthcare, as well as their interplay, has become clearer. Firstly, we briefly summarize the current status of three topics in this review: microbiome, TCM, and relationship of TCM and microbiome. Second, we focused on TCM's therapeutic effects and gut microbiome's mediation roles, including the relationships among diet, gut microbiome, and health care. Third, we have summarized some databases and tools to help understand the impact of TCM and gut microbiome on diagnosis and treatment at the molecular level. Finally, we introduce the effects of gut microbiome on TCM and host health, with two case studies, one on the metabolic effect of gut microbiome on TCM, and another on cancer treatment. In summary, we have reviewed the current status of the two components of healthcare: TCM and gut microbiome, as well as their concert effects. It is quite clear that as the holobiont, the maintenance of the health status of human would depend heavily on TCM, gut microbiome, and their combined effects.

## Introduction

With the rapid advancement of research in traditional Chinese medicine (TCM) and gut microbiome, studies involving both fields have significantly accelerated around the world. TCM and gut microbiome are closely related to human health. Therefore, exploring the relationship between TCM and gut microbiome is essential for healthcare.

### Microbiome

The microbiome is a novel research field related to human health, bioenergy, agriculture, and the environment. It has recently emerged as a crucial factor in human physiology and disease. Taking the microbiome into consideration, human beings could be considered “super organisms,” with around 10% human cells and 90% microbial cells, most of which can be found in the gut (Lederberg, 2000). The human gut is a biological niche, home to a variety of microbes that affect nearly all aspects of human biology through their interactions with their hosts (Blaser, 2014). The human intestinal flora is a complex micro-ecosystem, containing approximately 10^14^ microbes from more than 1,000 microbial species, of which the phyla *Firmicutes*, *Bacteroidetes*, and *Actinobacteria* (Gill et al., 2006) account for more than 90% of the species above. In addition, the genetic factors, dietary habits, and various environmental factors of the host lead to the diversity and the specificity of the human intestinal flora, while human intestinal flora in turn would play important roles in exerting host's physiological functions, such as metabolism, energy intake, and immune regulation.

Under normal circumstances, the intestinal flora is in a state of homeostasis. Environment, drugs, and the other factors that destroy the structure of intestinal flora will cause dysbiosis, which may influence human health. Recent studies have already accumulated ample evidences that the gut microbiome plays pivotal roles in many forms of chronic diseases. For example, inflammatory bowel disease (IBD) is a chronic bowel disease with clinical symptoms of abdominal pain, diarrhea, and unknown etiology, it includes ulcerative colitis and Crohn's disease (CD). The fecal microbiome of CD patients contains a significant reduction of *Firmicutes* diversity, and it's worth mentioning that the *Clostridium leptum* phylogenetic group has less abundance in CD patients than healthy (Manichanh et al., 2006). Constipation is a common health problem with high mortality, as well as a predisposing factor for many conditions. *Bifidobacterium adolescentis* exhibited strain-specific effects in alleviation of constipation (Wang et al., 2017). Obesity is a global problem in that nearly 12% of the people worldwide are obese. Obese mice had 50% fewer *Bacteroidetes* and more correspondingly *Firmicutes* than their lean littermates (Ley et al., 2005). Besides, they reached similar conclusions for obese people (Ley et al., 2007). Dietary interventions might lead to the modulation of gut microbiome, which will contribute to weight loss, enhance the integrity of the intestinal barrier, and reduce the antigenic load in the circulation, ultimately improving the inflammatory and metabolic phenotypes (Xiao et al., 2014). Type 2 diabetes (T2D) is a complex disorder affected by genetic and environmental components (Wellen and Hotamisligil, 2005; Risérus et al., 2009), and become a major public health problem on a global scale. A protocol has been developed for the metagenome-wide association study (MGWAS) and a two-phase approach has been taken based on high-throughput sequencing of the intestinal microbiome of 345 Chinese individuals. MGWAS analysis revealed that the intestinal microbiome of patients with T2D was mal-regulated, which was manifested by the decrease in the abundance of certain universal butyrate-producing bacteria and the increase of pathogenic bacteria in various conditions (Qin et al., 2012). Metformin is a widely used drug in the treatment of T2D. A recent study discovered the mechanism of metformin by regulating the composition of gut microbiome (Wu et al., 2017), indicating a close relation between the gut microbiome and T2D.

Human gut microbiome are structurally dynamic over time and plastic under different conditions, as the bacterial composition as well as the collection of genes varied with xenobiotics and the environment (Zhao et al., 2012). Due to the close relevance between the microbiome and the human genome, the taxonomic and functional composition changes of the intestinal microbiome inevitably regulate gene expression and host immunity, which may lead to the occurrence of diseases, in particular, chronic diseases.

In recent years, with the launching of various international projects on human microecology [e.g., HMP: http://www.hmpdacc.org/, MetaHit: http://www.metahit.eu/ and iHMP (Proctor et al., 2014)], gut microbiome have become the hotspot research between chronic diseases and the gut microbiome has steadily increased, promoting the most significant paradigm shifts in modern medicine.

### Traditional Chinese Medicine (TCM)

TCM is in constant development and inheritance along the long Chinese traditional culture. It has been developed in clinics over thousands of years and has accumulated abundant clinical experience, forming a field with unique experiences and specific theories. The TCM system is complex, as it contains components of natural plants, animals, and mineral materials. TCM uses therapeutic herbs to treat the disease and restore the balance of physical function according to the patient's syndrome, based on the combination principle of “King, Vassal, Assistant and Delivery Servant” (Yi and Chang, 2004). Each prescription combination of these herbs is called a TCM preparation or prescription, such as LiuWeiDiHuangWan (LWDHW) pills. Many Chinese herbal medicines are known for their therapeutic effects compared to chemical and biological agents (Chan, 1995; Corson and Crews, 2007; Qiu, 2007), which contain not only bioactive components but also various nutrients such as proteins and polysaccharides. For example, *Glycyrrhiza uralensis* Fisch., also called “Gan-Cao” in China, as a health-preserving and therapeutic agent, it has been widely utilized for more than 2,000 years. It is one of the most broadly used ingredients in TCM preparations in China, exerting a wide range of pharmacological efficacies. *Glycyrrhiza uralensis* Fisch. contains many bioactive ingredients, including glycyrrhizin, glycyrrhetinic acid, glycyrol, coumarin, and alkaloids. In addition, previous studies have shown that *Glycyrrhiza uralensis* Fisch. has a variety of pharmacological effects, including antitumor (Shibata et al., 1991), antiviral (Nakashima et al., 1987), and anti-inflammatory effects (Kimura et al., 1988).

### Relationship of TCM and Gut Microbiome

The traditional discovery pathway for most new drugs is to identify or design pharmacologically effective agents that specifically stimulate or inhibit a specific set of target receptors. Nevertheless, the influence of the gut microbiome on humans has been largely ignored in this process. Therefore, the pharmacological effects of these single target drugs on chronic diseases are limited. In contrast, since one of the characteristics of TCM is oral administration, the drug will interact with the intestinal flora inevitably. Previous studies have proven that TCM is conductive to maintain the homeostasis of the intestinal flora (Chang et al., 2015; Zhou et al., 2016), and the gut microbiome could also exert pharmacological effects of the TCM on host (Park et al., 2006), which render TCM a potential new drug in the western markets. Thus, research about relationship between TCM and gut microbiome is significant, which helps researchers to further study the pharmacological effects of TCM on the human body and the causal links among intestinal microbiome with disease.

**Figure 1:**
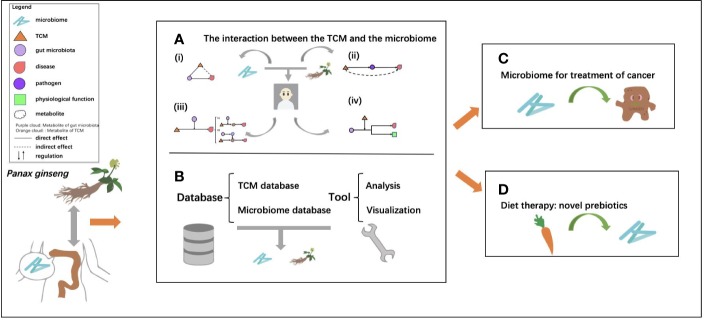
The general framework of the review. **(A)** The interactions between traditional Chinese medicine (TCM) and the microbiome, which are mainly divided into four types: (i) regulation effect of TCM on the microbiome, dysbiosis of intestinal flora is restored by TCM; (ii) TCM's broad-spectrum antibacterial effect to prevent the infection with pathogenic bacteria; (iii) mediation of gut microbiome on TCM, the main active ingredients of TCM are biotransformed by the microbiome; (iv) mediation effect of drugs and TCM on microbiome through gene regulation; **(B)** the related databases, including TCM databases and microbiome databases and tools for analysis and visualization for research of TCM and the microbiome; **(C)** microbiome as a key orchestrator for cancer treatment; and **(D)** food as a potential prebiotic for microbiome regulation.

In this review, we summarize the current status of microbiome, TCM, relationship of TCM and microbiome, as well as the therapeutic effects of TCM and gut microbiome's mediation roles. Among them, databases and tools that contribute to the molecular understanding of TCM and the role of the gut microbiome in diagnosis and treatment are also presented. Finally, we introduce the impacts of gut microbiome on TCM and host health, with two case studies, one on the metabolic effect of gut microbiome on TCM, and another on cancer treatment (**Figure 1**).

## Effects of TCM on Microbiome

**Figure 2:**
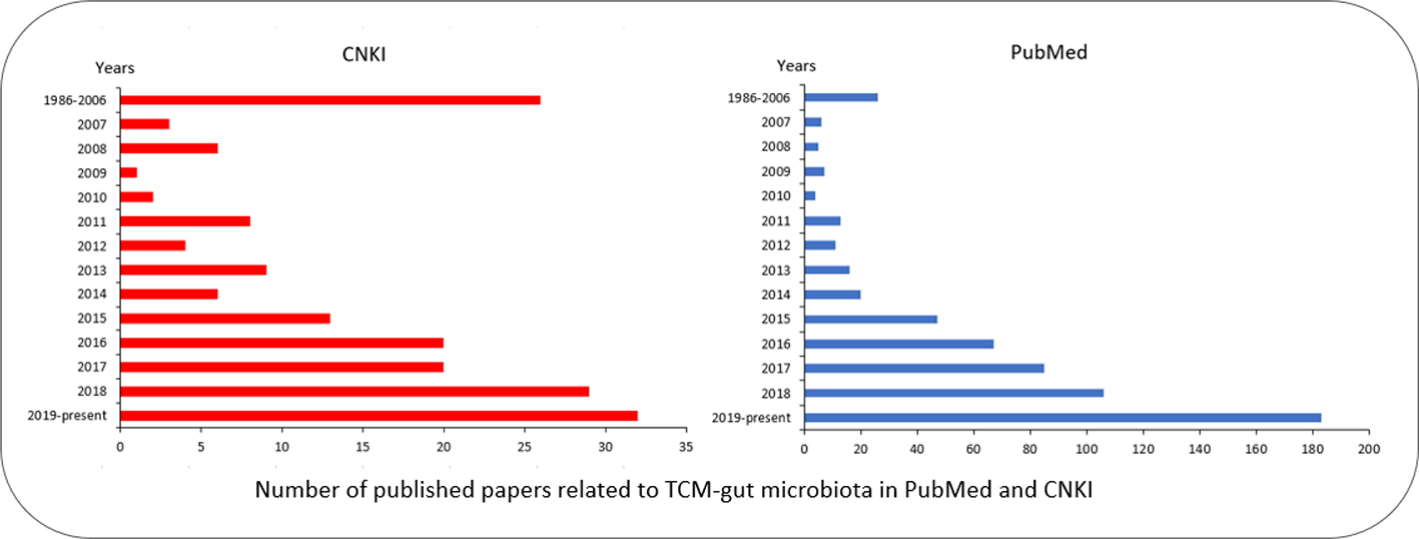
Number of published papers related to TCM-gut microbiota in PubMed and CNKI. Papers in PubMed were searched using keywords “gut/intestinal microbiota,” “Chinese,” and “medicine”; Papers in CNKI were searched by Chinese name of “TCM” and “gut microbiota/microbiome.” The red bar chart represents the papers in Chinese; the blue bar chart represents the papers in English.

An increasing number of studies have been conducted on TCM, gut microbiome, and their interplay. Statistics on the published papers in “Public Medicine (PubMed)” (https://pubmed.ncbi.nlm.nih.gov) and “China National Knowledge Infrastructure (CNKI)” (https://www.cnki.net) showed that the number of literature on TCM and gut microbiome continued to rise in recent years (**Figure 2**). Most importantly, due to the standardization of TCM, research on TCM and gut microbiome has received more and more attention around the world, and the TCM-related research outputs have shown explosive growth in 2019.

**Figure 3:**
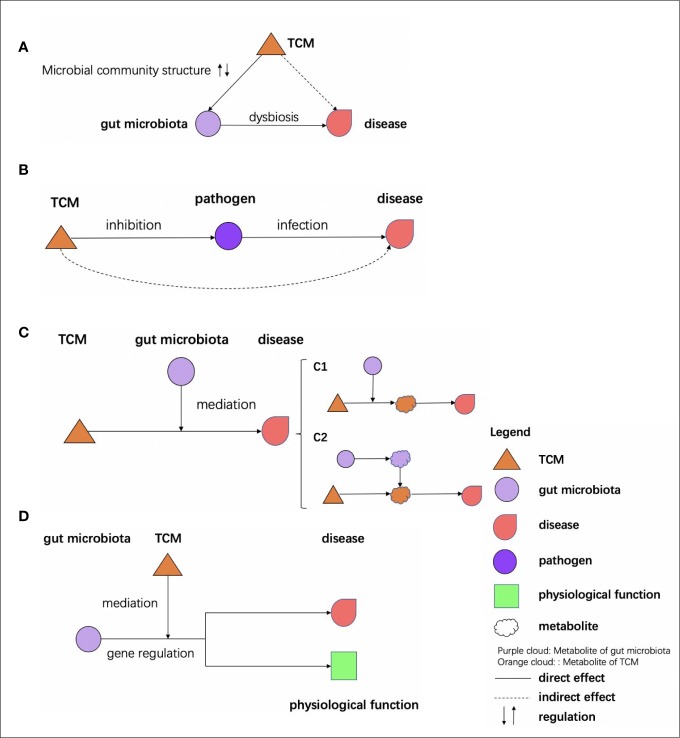
Effects of TCM on microbiome. **(A)** TCM affects the gut microbiota to restore dysbiosis. **(B)** TCM inhibits the pathogen to prevent the infection. **(C)** The bioactive ingredients of TCM are mediated by the gut microbiota to exert their effects: (c1) the bioactive ingredients of TCM are mediated by the gut microbiota (c2) the bioactive ingredients of TCM, and the metabolites of the gut microbiota would react and effect genes. **(D)** The gut microbiota effects are mediated by the TCM through gene regulation.

To maintain the health of humans under various physiological conditions and environments, TCM and the gut microbiome must coordinate with each other to cope with the challenges. The interactions between TCM and the gut microbiome can mainly be categorized into four types (**Figure 3**):

### Regulating Effects of TCM on Intestinal Flora and in Other Microbial Communities

The regulatory effects of TCM on gut microbial communities are mainly reflected in treatment of dysbiosis to restore homeostasis of the intestinal flora.

*Ganoderma lucidum* (*G. lucidum*), called “Ling-Zhi” in China, has been widely used as a health-preserving and therapeutic agent. A water extract of *G. lucidum* has antiobesogenic effects, as it could modulate the taxonomical composition of the gut microbiome (Chang et al., 2015). The water extract of *G. lucidum* mycelium can reverse high-fat diet (HFD)-induced gut dysbiosis, indicated by the decreased *Firmicutes-Bacteroidetes* ratios and endotoxin-bearing *Proteobacteria* levels. It can also maintain intestinal barrier integrity and reduce metabolic endotoxemia. In addition, it can reduce the expression and secretion of the proinflammatory cytokines tumour necrosis factor (TNF), interleukin-1-beta (IL-1β), and interleukin-6 (IL-6). The glucose tolerance and insulin sensitivity are improved by the extract as well. These effects are especially mediated by the gut microbiome, as it has already been observed from experiments on mice that the weight loss effect induced by *G. lucidum* is transmissible via transfer of feces from *G. lucidum*-treated mice to HFD-fed mice. The study has also demonstrated that the antiobesogenic effect of the *G. lucidum* water extract is mainly owing to its high molecular weight polysaccharide fraction (>300 kDa). *G. lucidum* polysaccharide can inhibit colon expansion of colorectal cancer (CRC) mice and reduce mortality by 30%, by alteration of reduced the relative abundance (RA) of cecal *Oscillospira*– which was first observed in CRC mice, and down-regulation of four cancer-related genes (Acaa1b, Fabp4, Mgll, and Scd1) expression of colonic epithelial cells (Luo et al., 2018).

Gegen Qinlian decoction (GQD), a traditional prescription, could effectively alleviate T2D by modulating the gut microbiome (Xu et al., 2013). In their study, compared to the patients with a low dose GQD and the placebo, patients who received high dose and moderate dose GQD showed remarkable reductions in adjusted mean changes from baseline fasting blood glucose (FBG) and glycated hemoglobin (HbA1c). *Faecalibacterium prausnitzii* was a species enriched significantly by GQD; the species was negatively correlated with HbA1c and FBG, while 2-h postprandial blood-glucose levels were positively correlated with homeostasis model assessment of β-cell function, which suggests that the structure of gut microbiome was changed by GQD. Furthermore, the amounts of beneficial bacterium, such as *Faecalibacterium spp*., could be enriched by GQD treatment. It has been reported that another TCM, *Polygonatum kingianum*, is effective in the treatment of diabetes and related diseases (Deng et al., 2012). A phytochemistry investigation demonstrated that the major types of active chemical constituents of *P. kingianum* were total saponins (TSPK) and total polysaccharides (PSPK). TSPK and PSPK could prevent the increase of fasting blood glucose after oral administration, and TSPK could increase the content of fasting insulin. Since TSPK and PSPK improved the intestinal microecology by increasing the abundance of *Firmicutes* and decreasing that of *Bacteroidetes* and *Proteobacteria*, TSPK and PSPK could prevent T2D by changing the regulation of the gut microbiome (Yan et al., 2017).

Huang-qin decoction (HQD) is widely used to alleviate gastrointestinal disorders such as inflammatory bowel disease. HQD significantly inhibited the weight loss, tissue damage, colon shortening, and inflammatory cytokine changes caused by dextran sulfate sodium (DSS). The relative abundance of *Lactococcus* in the DSS + HQP group was higher than that in the DSS group, while *Desulfovibrio* and *Helicobacter* were decreased, indicating that HQD can improve DSS-induced inflammation through its regulation in the gut microbiome. In addition to TCM directly acting on gut microbiome to ameliorate diseases, some TCMs exert indirect effects on the intestinal flora (Yang et al., 2017). Ginsenosides and polysaccharides in Du-Shen-Tang made from *Panax Ginseng* are used to investigate their pharmacological effects on acute cold stress and overfatigue. It was demonstrated that the intestinal metabolism and absorption of certain ginsenosides were improved by polysaccharides. In addition, in recovery of the disordered gut microbiome, polysaccharides especially boost the growth of two important probiotics, *Lactobacillus* spp. and *Bacteroides* spp. (Zhou et al., 2016).

*Glycyrrhiza uralensis* Fisch., also named licorice, has been commonly used for sore throat and gastrointestinal diseases. After administering different doses of licorice aqueous extracts to mice, they found that the proportion of *Bacteroides* gates decreased significantly, and the proportion of phylum *Firmicutes* increased and became dominant. *Bacteroides* was reported to be associated with IgA in humans and may cause colitis, and more phylum *Firmicutes* than *Bacteroides* lead to more efficient absorption of food calories. Therefore, it is speculated that licorice aqueous extract can promote intestinal absorption, anti-inflammatory effect, and treatment of abdominal pain (Xu et al., 2018). Diammonium glycyrrhizinate (DG), the main component of licorice root extracts, is a compound of the natural bioactive pentacyclic triterpenoid glycoside, can protect against nonalcoholic fatty liver disease (NAFLD) in mice through the decreasing of *Firmicutes*-to-*Bacteroidetes* ratio and endotoxin-producing bacteria such as *Desulfovibrio*, and elevate the abundance of probiotics such as *Proteobacteria* and *Lactobacillus*. DG can also augment the levels of short-chain fatty acid (SCFA)-producing bacteria such as *Ruminococcaceae* to promote SCFA production, and restoration of intestinal barrier (Li et al., 2018).

The regulation of TCM on human health can also be reflected in other microbial communities, including saliva and tongue coating (Jiang et al., 2012; Liang et al., 2014; Li, 2015). For instance, through a network pharmacology approach (Li, 2015), the tongue coating microbiome have been found to be associated with various diseases including colorectal cancer (Liang et al., 2014) and “cold-disease” (Jiang et al., 2012). Therefore, the regulation of TCMs can be reflected in microbial communities of various human body habitats, including gut and oral habitats, indicating quite diverse microbial-based approaches for noninvasive probing of human health status.

### Inhibition of Pathogens by TCM

In recent years, with the widespread use of synthetic and semisynthetic antibiotics, the problem of bacterial resistance has become increasingly serious in clinical fields. Pathogenic bacterial infections are critical factors that may affect the development and severity of the disease. TCM was reported to possess a broad-spectrum antibacterial effect. Therefore, systematic study on the antibacterial activity of TCM and the further development of new drugs have become the focus of more and more researchers.

Currently, more and more research indicate that TCM has antibacterial effect (**Table 1**).

**Table 1:** Antibacterial effects of traditional Chinese medicine (TCM).

Herb	Bacteria	Reference
*Euphorbia humifusa* Willd.	*Staphylococcus aureus* *Staphylococcus albus* *Pseudomonas aeruginosa* *Escherichia coli* *Salmonella typhi* *Streptococcus B*	(Bai et al., 2007)
*Magnolia officinalis* Rehd. *et* Wils.	*Staphylococcus aureus* *Staphylococcus albus* *Pseudomonas aeruginosa* *Escherichia coli* *Salmonella typhi* *Streptococcus B*	(Wang et al., 2007)
*Nerium indicum* Mill. *Mangifera indica* *Melia azedarace* L. *Bauhinia purpurea* Linn.	*Vibrio*	(Guan et al., 2008)
*Coptis chinensis* Franch. *Rheum officinale* Baill. *Forsythia suspensa* *Sophora flavescens* *Rehmanniae Radix* *Anemarrhena asphodeloides Bunge*	*Neissenria gonorhoea*	(Yang et al., 2009)
*Sophora alopecuroides* L.	*Staphylococcus epidermidis*	(Wan et al., 2015)
*Houttuynia cordata* Thunb.	*Mycobacterium smegmatis* *Streptococcus mutans* *Staphylococcus aureus* *Enterococcus faecalis* *Candida albicans*	(Verma et al., 2017)
*Phellodendron lavallei*	*Staphylococcus aureus* *Enterococcus faecalis* *Escherichia coli* *Klebsiella pneumoniae*	(Lis et al., 2017)
*Portulaca oleracea* L.	*Staphylococcus aureus*	(Fung et al., 2017)
*Sophora flavescens*	*Escherichia coli* *Staphylococcus aureus*	(Zhang et al., 2018)
*Brassica rapa* L.	*Clostridium perfringens*	(Wang et al., 2018)
*Lonicera japonica* Thunb.	*Escherichia coli* *Micrococcus luteus* *Bacillus cereus* *Staphylococcus aureus*	(Yang et al., 2018)
*Taraxacum mongolicum Hand*. Mazz.	*Aeromonas hydrophila* *Bacillus subtilis* *Bordetella bronchiseptica* *Enterococcus faecalis* *Escherichia coli* *Klebsiella pneumoniae* *Micrococcus luteus* *Pseudomonas aeruginosa* *Salmonella typhimurium* *Serratia marcescens*	(Sharifi-Rad et al., 2018)
*Curcuma longa* L.	*Staphylococcus aureus sub sp*. *Bacillus subtilis*	(Irshad et al., 2018)
*Coptidis Rhizoma*	*Streptococcus agalactiae* *Actinobacillus pleuropneumoniae* *Staphylococcus aureus* *Coagulase-negative Staphylococcus* *Escherichia coli* *Helicobacter pylori* *Salmonella Typhimurium* *Candida albicans*	(Wang et al., 2019)
*Panax ginseng*	*Staphylococcus aureus* *Escherichia coli* *Bacillus pumilus* *Bacillus subtilis*	(Zhao et al., 2019)
*Glycyrrhiza uralensis* Fisch.	*Streptococcus mutans*	(Yamashita et al., 2019)
*Forsythia suspensa* *Lonicera japonica* Thunb. *Ephedra sinica Stapf* *Semen Armeniacae* Amarum. *Isatisindigotica* Fort. *Dryopteridis Crassirhizomatis Rhizoma* *Houttuynia cordata* Thunb. *Gypsum Fibrosum* *Pogostemon cablin* Benth. *Rhei Radix et Rhizoma* *Rhodiola rosea* L. *Mentha canadaensis* L. *Glycyrrhiza uralensis* Fisch.	SARS-novel *Coronavirus*-2	(Li et al., 2020a)

These antibacterial effects correspond to various diseases, including digestive diseases (sorted by year).

### Close Relationships Among Diet, Gut Microbiome, and Health Care

In the long history of TCM treatment, diet has always been considered as an important category of resources. Development of the research of gut microbiome involves not only the drug and TCM but also the daily diet of humans. Recently, a rapidly increasing number of studies have indicated the crucial role of the diet for the treatment of disease (Leboeuf et al., 2020; Li et al., 2020b; Moayyedi et al., 2020; Naudin et al., 2020; Yu et al., 2020). In addition, studies about the close relationship between the diet and the intestinal flora have gradually aroused attention, which may offer us some new perspective for disease prevention.

Carbohydrates are important components of the daily diet and include monosaccharides, disaccharides, and polysaccharides. Both fructooligosaccharide (FOS) and polyphenols can be transferred directly into the large intestine of mammals alone, reshaping the composition of gut microbiome, which is beneficial to human health. The combination of different phenolic compounds and FOS had a distinct impact upon the host, and gut microbiome improved by using prebiotics can delay the onset of senescence-related health problems (Zheng et al., 2017; Tanabe et al., 2019). Adding catechin to an FOS diet could inhibit *Firmicutes* and enhance *Bacteroidetes*. In addition, it turns out that antibiotic treatment influences the diversity and composition of the gut microbial community. Furthermore, the use of probiotics or prebiotics to modulate antibiotic-induced gut microbiome destruction has been considered a desired treatment or prophylaxis (Bron et al., 2011). Cefixime can reduce the diversity of the microbial community and lead to a significant decrease in the relative abundance of *Firmicutes*. The composition of gut microbiome of *Lactobacillus* and FOS probiotic mixture treatment group was much more diverse than that of the natural recovery group, indicating a better recovery effect of the probiotic cocktails on the gut microbiome (Shi et al., 2017). Moreover, the composition of the gut microbiome significantly changed in the HFD + fructose (HFF)-fed and the HFD + sucrose (HFS)-fed rats compared with the control diet (C)-fed rats; body-fat mass, metabolic inflexibility, glucose intolerance, lipopolysaccharide (LPS), insulin, renal reactive oxygen species (ROS), malondialdehyde (MDA), Nadphox, and Srebp-1 were significantly higher, and antioxidant enzymes and lean body masses were significantly lower in the HFS group with respect for the HFF group (Rosas-Villegas et al., 2017), indicating the harmful effect on the HFS group and the HFF group on gut microbiome as well as the health of humans. Carrageenan, agarose, and alginate are algae-derived undigested polysaccharides that have been used as food additives for hundreds of years. *Bacteroides* uniforms L8 could degrade agarose completely, and the major enzyme secreted was β-agarase. The enzyme produced by isolate 38F6 (*Bacteroides xylanisolvens* and *Escherichia coli*), which degrades κ-carrageenan oligosaccharides, is β-carrageenase, and alginate, guluronic acid oligosaccharides, and mannuronic acid oligosaccharides could be degraded by two enzymes from *Bacteroides ovatus* G19 (Li et al., 2017).

Vitamin D is an important pro-hormone for optimal intestinal calcium absorption for mineralization of bone (Khazai et al., 2008), which is common in high-fat food such as cream and salmon. Vitamin D deficiency is a disease caused by air pollution, insufficient sunlight exposure, and altered dietary composition, which have also been associated with other diseases, including autoimmune diseases, hepatitis, and cancer (Holick et al., 2011). The functionality of the vitamin D-vitamin D receptor (VDR) axis in up-regulating and the activation/process of ileal α-defensins was identified. Conversely, dietary vitamin D deviancy resulted in the loss of Paneth cell-specific α-defensins, which may consequently lead to intestinal dysbiosis and endotoxemia, indicating that vitamin D is essential for the homeostasis of intestinal flora (Su et al., 2016).

Acetate has been widely distributed in nature, such as in fruits or vegetable oils. A recent study showed that acetate produced by protective *Bifidobacteria* could improve intestinal defense that is mediated by epithelial cells, and this could protect the host against negative infection (Fukuda et al., 2011). In addition, moderate consumption of wine has shown the potential to delay the onset of neurodegenerative diseases: After drinking, microbial metabolites may effectively protect neuroblastoma cells from nitrosative stress injury (Esteban-Fernandez et al., 2017).

## Tools and Databases

**Figure 4:**
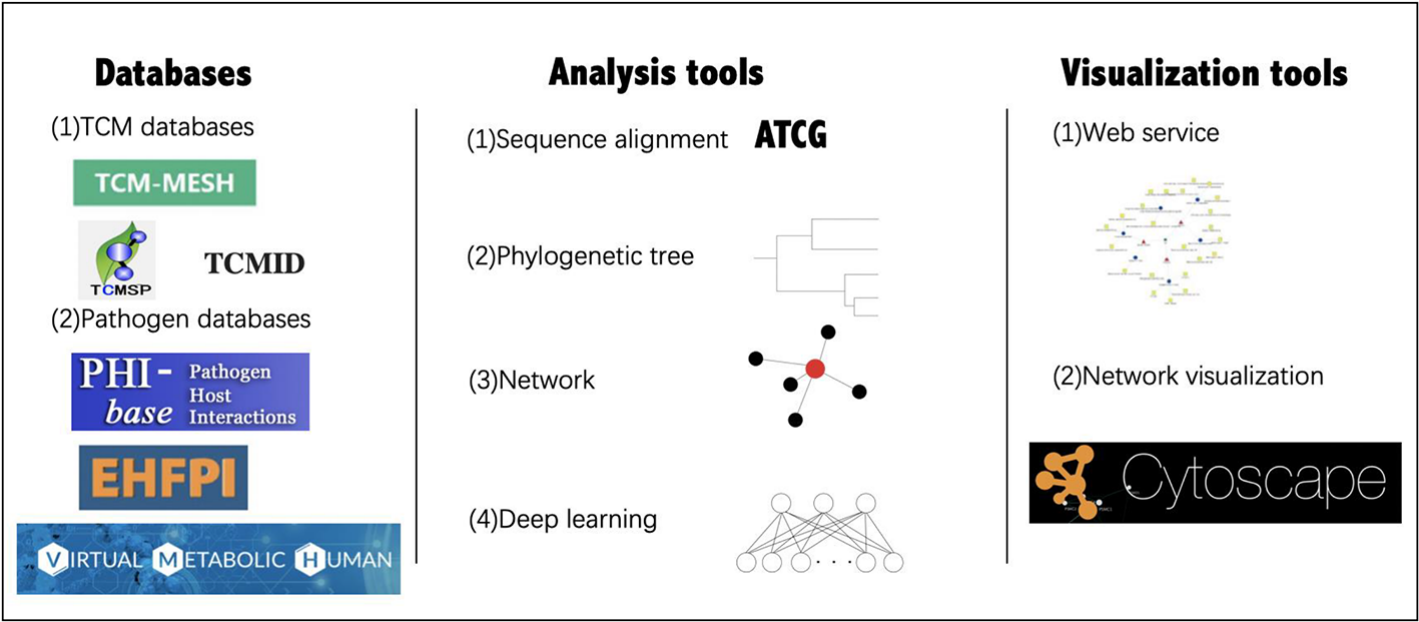
Databases and tools for TCM-gut microbiome research. The databases and tools in the figure are cited in the following section.

With the application of network pharmacology and related databases and tools, the research on TCM and gut microbiome has been further developed (**Figure 4**).

Commonly used databases of TCM gut microbiome research include the TCM database [TCM-Mesh (Zhang et al., 2017), TCMSP (Ru et al., 2014), TCMID (Xue et al., 2013), and HIT (Ye et al., 2011)], as well as pathogen-host databases [PHI-base (Winnenburg et al., 2006) and EHFPI (Liu et al., 2015)]. TCM-Mesh has integrated information about TCM ingredients, compound-protein interactions, as well as protein-disease relationships, to establish the largest resource for TCM target genes and diseases. Based on the biological databases and clinical trial results, a researcher can analyze the “TCM-host-gut microbiome” interaction network from a holistic perspective. Virtual Metabolic Human database (Noronha et al., 2018) contains information on human and gut microbial metabolism, which is linked to hundreds of diseases and nutritional data by mathematical models.

Many tools are usually used for analysis and visualization as well. For example, QIIME (Caporaso et al., 2010) is a software that provides an open-source bioinformatics pipeline for performing microbiome analysis from raw DNA sequencing data. It includes demultiplexing, quality filtering, operational taxonomic units (OTU) picking, taxonomic assignment, phylogenetic reconstruction, diversity of analyses and visualizations. PICRUSt (Langille et al., 2013) is designed to predict microbiome functional content from marker gene (e.g., 16S rRNA) surveys and full genomes. MetaPhlAn (Segata et al., 2013) is a computational tool for microbial communities analysis based on metagenomic sequencing data, in which the data were compared with the markers of each species obtained from the known database to determine the species category. MUSCLE (Edgar, 2004) and CLUSTALW (Thompson et al., 1994) are two algorithms used for multiple sequence alignments; MUSCLE is a computer program for creating multiple alignments of protein sequences. MEGA (Kumar et al., 2016) is a computer program package for estimating evolutionary distances, reconstructing phylogenetic trees, and computing basic statistical quantities from molecular data. Additionally, iTOL (Letunic and Bork, 2016) is an online tool for visualization, annotation, and management of phylogenetic trees. Furthermore, with the development of deep learning, many methods have been used for studying the gut microbiome and diseases. For example, a model was constructed to predict responses to anti-integrin biologic therapy for inflammatory bowel diseases through gut microbiome function (Ananthakrishnan et al., 2017). Although many approaches are not currently used in TCM study on gut microbiome, related methods will become a future trend. Therefore, additional investigations are warranted.

Furthermore, interactive visualization tools are of equal importance. Cytoscape is an open-source platform that could be used for complex network analysis and visualization (Shannon et al., 2003), which is useful for analyzing the network in network pharmacology studies. Similar functions are also offered by the web services of many databases.

## Gut Microbiome: A Key Player Mediating TCM and Other Medicinal treatments

The involvement of the gut microbiome in maintaining the health status of every person will undoubtedly affect the effectiveness of drug treatments, including TCM treatment. Here, we provide two case studies to illustrate this, one on the metabolic effect of gut microbiome on TCM, and the other on cancer treatment, to illustrate the mediation role of the gut microbiome in medicinal treatment in general.

### Metabolic Effect of Gut Microbiome on TCM

Previous studies have found that many of the chemical constituents of TCM can be transformed and metabolized by the intestinal flora, making it easier for the body to absorb. In other words, the gut is like a processing factory of TCM. The effects of the intestinal flora on different kinds of chemical constituents vary:

#### Biotransformation of Flavonoids

Flavonoids represent a major class of secondary metabolites of plants and can be classified into 12 subclasses: anthocyanins, chalcones, dihydrochalcones, flavanols, flavanones, flavones, flavonols, isoflavones, flavan-3-ols, flavan-3,4-diols, aurones, and proanthocyanidins (Crozier et al., 2009). The effects of these polyphenols in the prevention of cancer, cardiovascular disease, T2D, and cognitive dysfunction are supported by an increasing number of studies (Del et al., 2013; Rodriguez-Mateos et al., 2014). To exert their effects, flavonoids are metabolized by the gut microbiome, and the resulting compounds may have bioactivity. Such bacterial conversion and potential health consequences for the human host (Chiou et al., 2014) are hardly unique: Baicalin, puerarin, and daidzin, which are widely found in TCM prescriptions, can also be metabolized by gut microbiome (Kim et al., 1998). As baicalin is difficult to be absorbed in the gut, it has to be hydrolyzed by gut microbiome to baicalein to exert its effect. Metabolism of baicalin was hardly detected in germ-free rats compared to conventional rats, indicating that gut microbiome play a key role in the metabolism of baicalin (Akao et al., 2001).

#### Biotransformation of Saponins

Saponins can be categorized into steroidal saponins and triterpenoid saponins according to the structure of the aglycone and are widely distributed in nature. Commonly used TCMs, including *Panax ginseng* C. A. Mey., *Astragalus membranaceus* (Fisch). Bunge, *Glycyrrhiza uralensis* Fisch., and *Ophiopogon japonicus* (Linn. f.) Ker-Gawl. are enriched in saponins. Ginsenosides belong to triterpenoid saponins and are the main active chemical ingredients of *Panax ginseng*. Currently, pharmacological investigations indicate that ginsengosides have an extensive range of biological activities, such as anti-inflammatory, antitumor and antidementia effects (Mochizuki et al., 1995; Sun et al., 2008; Shi et al., 2013). Ginsenoside compounds K (CK) is one of the metabolites of ginsengoside, which exerts various pharmacological effects, including antiallergic (Shin and Kim, 2005), antidiabetic (Kim et al., 2014), anticarcinogenic (Zhou et al., 2010; Zhang et al., 2013), anti-inflammatory (Joh et al., 2011; Chen et al., 2014), and antiaging effects (Kim et al., 2004). The occurrence of CK in rat plasma is required for intestinal bacterial hydrolysis after oral administration of ginsengosides (Akao et al., 1998). When human intestinal flora was cultured anaerobically with ginsengosides Rb1, Rb2, and Rc, these ginsengosides were metabolized into CK, where the transformation of Rc is conducted by the concert efforts of *Bacteroides* spp., *Eubacterium* spp., and *Bifidobacterium* spp. (Bae et al., 2000; Bae et al., 2002). In addition, intestinal bacterial metabolism of ginsengosides was mainly dependent on the composition of intestinal flora, such as *Bacteroides* spp., *Ruminococcus* spp., and *Bifidobactetium* spp. (Kyung-Ah Kim et al., 2013). Additionally, the metabolism of panax notoginseng saponins (PNSs) was influenced by gut microbiome, of which proteobacteria may have an effect on the deglycosylated metabolism of PNSs by regulating the activities of glycosidases (Xiao et al., 2016). Up-regulation of *Bacteroidetes* may increase the activity of redox metabolic enzymes in intestinal flora and accelerate the redox metabolism of PNSs (Xiao et al., 2016).

#### Biotransformation of Alkaloids

Oxymatrine and matrine are two main alkaloids in the radix *Sophorae flavescentis*, and Oxymatrine could be transformed into matrine by intestinal bacteria when taken orally, and both can be absorbed by the blood (Wang et al., 2001). Scopolamine was incubated with rat intestinal flora *in vitro* under limited oxygen conditions, and scopolamine was transformed by gut microbiome into scopine (Chen et al., 2006).

#### Biotransformation of anthraquinones

Anthraquinones have widespread applications throughout industry and medicine, and are naturally distributed in many plants, including *Rheum officinale* Baill., *Fallopia multiflora* (Thunb.) Harald., *Catsia tora* Linn., *Folium Sennae*, and *Aloe vera* (Linn.) N. L. Burman var*. chinensis* (Haw.) Berg. Through a chemical constituent study on the metabolism of rhubarb extract by rat intestinal bacteria, a total of 14 components, including emodin-*O*-glucosides, aloe-emodin-*O*-glucosides, physcion-*O*-glucosides, chrysophanol-*O*-glucosides, and their aglycones were found to be biotransformed by rat intestinal bacteria. Twelve major metabolites were identified in the incubation sample, suggesting the importance of the intestinal flora during the metabolism of anthraquinones (Song et al., 2011).

### Microbiome: A Key Orchestrator for Cancer Treatment

Carcinogenesis may be mediated by microbiome influencing cellular metabolism, inflammation, and proliferation (Zitvogel et al., 2017). The microbiome regulate cancer at the level of predisposing conditions, initiation, genetic instability, susceptibility to host immune response, progression, comorbidity, and response to therapy. Several examples are as follows:

There is an increasing number of studies involving mice and humans on the role of intestinal flora in cancer treatments. In mice, the procarcinogenic phenotype expressed by genetically mutated mice has been shown to be transferred to wild-type mice by microbiome transfer (Garrett et al., 2009; Couturier-Maillard et al., 2013; Hu et al., 2013), and transfer of fecal microbiome from patients who are sensitive to cancer treatment into germ-free mice has been found to endow these animals with an ability to respond efficiently to therapy (Vetizou et al., 2015), indicating that changes in the composition of intestinal flora indirectly affect carcinogenesis and response to cancer treatment through lifestyle and host genetic.

Furthermore, the intestinal flora affects anticancer activity, toxicity, as well as pharmacokinetics of drugs at various levels (Dzutsev et al., 2015; Spanogiannopoulos et al., 2016). Several anticancer drugs have experimentally been shown to be affected by the intestinal flora (Haiser and Turnbaugh, 2013), including the hydrolysis of methotrexate, the nitroreduction of misonidazole, and the deconjugation of irinotecan. In germ-free mice or mice in which gut commensals were exhausted by broad-spectrum antibiotics, the antitumor effect of cisplatin or oxaliplatin treatment on subcutaneous transplantable tumors was decreased evidently (Lida et al., 2013). Probiotics *Lactobacillus acidophilus* in antibiotic-treated mice restored cisplatin-induced inflammatory gene expression (Gui et al., 2015), indicating the mediation role of gut microbiome on drug efficacy. Additionally, oral administration with *Bifidobacterium bifidum* and *L. acidophilus*, a probiotic combination, was found to prevent intestinal toxicity in cancer patients who were treated with both cisplatin and radiotherapy (Chitapanarux et al., 2010). In addition to the direct effects on the enzymes and microbiome on the absorption and metabolism of drugs (Maurice et al., 2013; Feng et al., 2015; Montassier et al., 2015), the indirect effects of gut microbiome on the metabolism of drugs were reported to occur through regulation of gene expression and the physiological function of the local mucosal barrier and of distant organs, such as the liver (Bjorkholm et al., 2009; Selwyn et al., 2015; Selwyn et al., 2016).

Radiotherapy is one of the traditional methods for cancer treatment. Because radiotherapy alters the microbiome composition and destroys the intestinal barrier (Barker et al., 2015), pathobionts can easily access the intestinal immune system (Belkaid and Hand, 2014). With the development of research on cancer and microbiome, probiotics have been reported to be beneficial in prevention of radiation-induced enteropathy in some clinical studies. For example, patients with head and neck cancer received *Lactobacillus brevis* CD2 lozenges during chemotherapy and radiation, and the incidence of treatment-induced mucositis decreased while the treatment completion rate increased (Sharma et al., 2012). Immunotherapy is another approach that has shown potential in treating solid cancers and hematopoietic (Couzin-Frankel, 2013). The proliferation and antitumor cytotoxic activity of transferred T cells in the tumor were increased by the total-body irradiation-induced translocation of intestinal flora into the mesenteric lymph nodes (Paulos et al., 2007).

Moreover, many studies have revealed the crucial role of intestinal flora in colorectal cancer treatment recently (**Table 2**), indicating the close relationship between gut microbiome and cancer treatment.

**Table 2:** Recent studies on the close relationship between colorectal cancer (CRC) and the gut microbiome.

Description	Year	Reference
Fecal metabolomic signatures are associated with gut microbiome and colorectal cancer pathogenesis: (1) The levels of polyunsaturated fatty acids and sphingolipids are further increased in patients with CRC, which may represent early changes in CRC. (2) Regardless of disease status, age, or gender, there is a strong overall correlation between microbiome and metabolomics data, and the correlation is higher in women than in men. (3) The abundance of multiple bacteria in *Firmicutes*, *Actinobacteria*, and *Bacteroides* is strongly correlated with elevated metabolite pathways in patients.	2020	(Kim et al., 2020)
Non-nucleatum *Fusobacteria* common in southern Chinese populations are associated with colorectal cancer: (1) Irrespective of CRC disease status, the prevalence, relative abundance, and diversity of *Fusobacterium* in southern Chinese populations are higher than those in other regions. (2) In addition to non-nucleatum *Fusobacteria*, *Fusobacterium* varium and *Fusobacterium* ulcerans and other *Fusobacterium* groups are also enriched in CRC patients. (3) Several non-nucleatum taxa possess FadA homologues and were enriched in CRC cohorts.	2020	(Yeoh et al., 2020)
A pathological imbalance of the gut microbiome (dysbiosis) is present in colorectal cancer patients: (1) Bacteria affect CRC directly or indirectly, by secreting metabolites, by invading tissues, and by modulating the host immune response. (2) *Fusobacterium*, *Peptostreptococcus*, *Porphyromonas*, *Prevotella*, *Parvimonas*, *Bacteroides*, and *Gemella* are among the most prominent CRC-associated bacteria.	2020	(Ternes et al., 2020)
Changes in gut microbiome can reduce the carcinogenic effect of colitis-related colorectal cancer: (1) *Enterobacter* strains are associated with molybdenum-dependent metabolic pathways in the colitis model. Oral sodium tungstate can inhibit the activity of *E. coli* molybdenum and reduce the colonization of *E. coli* in the intestine. (2) Limiting the growth of *Enterobacteriaceae* can control intestinal inflammation and reduce the incidence of colorectal cancer. (3) Oral sodium tungstate treatment can reduce intestinal inflammation and inhibit tumorigenesis caused by colicin.	2019	(Zhu et al., 2019)
Colorectal cancer can be diagnosed by *Lachnoclostridium* as a marker: (1) Metagenomic analysis identified “m3” from a *Lachnoclostridium* sp., *Fusobacterium nucleatum* (Fn), and *Clostridium hathewayi* (Ch) to be significantly enriched in CRC. (2) The combination of m3 with Fn, Ch, *Bacteroides*, and FIT can diagnose CRC most accurately.	2019	(Liang et al., 2019)
Intestinal fungal disorders in colorectal cancer, expected to be used for diagnosis: (1) CRC intestinal fungal disorders, increased ratio of *Basidiomycetes/Ascomycetes*, increased *Malassezia*, decreased yeast and *Pneumocystis*, 6 genus enrichment, 38 species abundance changes. (2) The co-occurrence association between fungi and the mutual exclusion of bacteria-fungi in CRC increased, and the positive interaction between *Proteobacteria* and *Ascomycota* became mutually exclusive in CRC.	2019	(Coker et al., 2019)
Different intestinal flora species increased or decreased the effectiveness of drugs: (1) Bacterial nucleotide metabolism genes in *Caenorhabditis elegans* affect drug efficacy. (2) 5-fluorouracil and 5-fluoro-2'-deoxyuridine act through bacterial ribonucleotide metabolism to exert their cytotoxic effects in *C. elegans* rather than by thymineless death or DNA damage.	2017	(Garcia-Gonzalez et al., 2017)
Two-way bacterial mediation effects of fluoropyrimidine on host metabolism may contribute to drug efficacy: (1) Microbes can bolster or suppress the effects of fluoropyrimidines through metabolic drug interconversion involving bacterial vitamin B_6_, B_9_, and ribonucleotide metabolism. (2) Disturbances in bacterial deoxynucleotide pools amplify 5-FU-induced autophagy and cell death in host cells.	2017	(Scott et al., 2017)
Levels of *P. anaerobius* are higher in human colon tumor tissues and adenomas compared with nontumor tissues; this bacterium increases colon dysplasia in a colorectal cancer mouse model. *P. anaerobius* interacts with TLR2 and TLR4 in colon cells to increase the abundance of reactive oxidative species, which promotes cholesterol synthesis and cell proliferation.	2017	(Tsoi et al., 2017)
*F. nucleatum* plays a key role in colorectal carcinogenesis through suppression of the hosts' immune response to tumor. A diet rich in whole grains and dietary fiber is associated with a lower risk of *F. nucleatum*-positive colorectal cancer.	2017	(Mehta et al., 2017)
Key microbial markers are critical in the classification of CRC cases and are commonly used in the diagnosis of disease.	2017	(Shah et al., 2017)
(1) AIM2-deficient mice are hypersusceptible to colonic tumor development. (2) Susceptibility of AIM2-deficient mice to colorectal tumorigenesis is enhanced by dysbiotic gut microbiome compared with healthy wild-type mice.	2015	(Man et al., 2015)

## Conclusions and Perspectives

In recent years, with the modernization of TCM and the development of systems biology, our understanding of TCM has been significantly advanced. TCM has gradually transformed from an experience-based approach to an evidence-based medicinal system, of which Chinese scientists have made great contributions. We modify this sentence to “For example, it was discovered that arsenic induces the degradation of a key leukemogenic protein and exerts a pharmacological effect on the treatment of acute promyelocytic leukemia (Zhang et al., 2001). Youyou Tu, the Nobel Prize winner, has also focused on TCM research and research on the combination of western medicine and TCM for many years (Tu et al., 1981; Tu et al., 1982). The trend has become clear that more and more TCM researches use molecular biology approach. However, with the systems biology approach enabled, the potential of TCM studies at the molecular level remains to be better exploited.

On the other hand, research about the microbiome, in particular the gut microbiome, has made enormous progress during recent years. With the launch of various international projects on the human microbiome, research on the gut microbiome has become a hot research area. Studies have proven that TCM could be used as a perfect agent to treat many kinds of diseases, many of which were mediated by gut microbiome. Therefore, combination of the research of TCM and gut microbiome is important to maintain the healthy status of the host-microbiome holobiont. In addition, with the advancement of biological big data research, databases can now provide a more holistic perspective for TCM research and intestinal flora research. However, with the regulation or mediation principles of gut microbiome on human health still lacking except for a few diseases, more microbiome experiments, sequencing data, and analytical methods have yet to be conducted, collected, or developed to better understand these principles.

It has become urgent that several problems remain to be solved for both TCM and gut microbiome research areas, especially in the era of biological big-data. With the constant increase in research in related fields, various experimental data have been generated. These all require better ways to curate, analyze, and interpret the concerted effects of TCM and the microbiome on human health. First, a more comprehensive database on TCM and gut microbiome is needed, which should not only include the interaction between the TCM and the gut microbiome but should also represent the advantages of TCM databases and microbiome databases in accelerating the application of TCM on a global scale. Second, analytical methods must keep pace with the rapid development of modern systems biology. A more powerful data mining tool is required to investigate the complex and multi-scale “TCM-host-microbiome” network. Third, investigations on the mechanisms of mutual regulation between TCM and gut microbiome are limited. How do various TCMs regulate dysbiotic microbiome in concert? What kinds of enzymes produced by gut microbiome are responsible for metabolizing TCM and exerting the effect of TCM? These only represent a few questions that are to be answered. Fourth, the microbiome has been indicated as a precision medicine frontier (Kuntz and Gilbert, 2017), as interindividual differences in microbiome patterns have been reported, even between co-raised twins. Finally, it is only when enough time-series samples are collected can we answer questions about dynamic patterns for the “TCM-host-microbiome” network. Such dynamic pattern is the basis for the “shelf life” and “personalized medicine” for the microbiome-mediated TCM treatment, and could push for the modernization of TCM.

In summary, researchers might take advantage of TCM and microbiome for better health care and treatment, as both possess the great potential in health care. This might lead to another paradigm shift: from genome-centric precision medicine to systems biology approach enabled holobiont-centric precision medicine.

## Author Contributions

KN and HB conceived and proposed the idea. KN, HB, and RZ jointly designed the main idea of this work. KN, RZ, and XG contributed to the interpretation of data for the work. KN, HB, XG and RZ drafted the manuscript, KN, HB, XG, and RZ revised it critically for important intellectual content. All authors read and approved the final manuscript to be published, and agree to be accountable for all aspects of the work in ensuring that questions related to the accuracy or integrity of any part of the work are appropriately investigated and resolved.

## Funding

This work was partially supported by National Science Foundation of China grant 81774008, 81573702, 31871334, and 31671374, and the Ministry of Science and Technology (High-Tech) grant (No. 2018YFC0910502).

## Conflict of Interest

The authors declare that the research was conducted in the absence of any commercial or financial relationships that could be construed as a potential conflict of interest.
